# Burden of disease and costs of aneurysmal subarachnoid haemorrhage (aSAH) in the United Kingdom

**DOI:** 10.1186/1478-7547-8-6

**Published:** 2010-04-27

**Authors:** Oliver Rivero-Arias, Alastair Gray, Jane Wolstenholme

**Affiliations:** 1 Health Economics Research Centre, Department of Public Health, University of Oxford, UK

## Abstract

**Background:**

To estimate life years and quality-adjusted life years (QALYs) lost and the economic burden of aneurysmal subarachnoid haemorrhage (aSAH) in the United Kingdom including healthcare and non-healthcare costs from a societal perspective.

**Methods:**

All UK residents in 2005 with aSAH (International Classification of Diseases 10^th ^revision (ICD-10) code I60). Sex and age-specific abridged life tables were generated for a general population and aSAH cohorts. QALYs in each cohort were calculated adjusting the life tables with health-related quality of life (HRQL) data. Healthcare costs included hospital expenditure, cerebrovascular rehabilitation, primary care and community health and social services. Non-healthcare costs included informal care and productivity losses arising from morbidity and premature death.

**Results:**

A total of 80,356 life years and 74,807 quality-adjusted life years were estimated to be lost due to aSAH in the UK in 2005. aSAH costs the National Health Service (NHS) £168.2 million annually with hospital inpatient admissions accounting for 59%, community health and social services for 18%, aSAH-related operations for 15% and cerebrovascular rehabilitation for 6% of the total NHS estimated costs. The average per patient cost for the NHS was estimated to be £23,294. The total economic burden (including informal care and using the human capital method to estimate production losses) of a SAH in the United Kingdom was estimated to be £510 million annually.

**Conclusion:**

The economic and disease burden of aSAH in the United Kingdom is reported in this study. Decision-makers can use these results to complement other information when informing prevention policies in this field and to relate health care expenditures to disease categories.

## Introduction

Aneurysmal subarachnoid haemorrhage (aSAH) (International Classification of Diseases 10^th ^revision code I60) is a type of cerebrovascular disease and a main cause of disability and mortality in relatively young patients, with an average age at first onset of 55 [1]. The incidence of aSAH has been estimated at around 6-7 per 100,000 people in most populations [1]. The epidemiology and effectiveness of treatments of aSAH is well-documented in the literature [2] and cost analyses of alternative therapies to treat aSAH are also available [3-5]. However the overall economic burden of aSAH to society remains unknown.

Making accurate economic estimates of resources associated with particular health problems provides useful information for Departments of Health worldwide [6]. These figures can be used by health care decision makers to understand the overall impact of a disease on the annual health care budget and to provide parameter estimates for economic models, including value of information studies. If performed at regular intervals such studies help to monitor the impact of health care policies as well as changes in clinical practice. For example, in the field of aSAH, the increased use of endovascular intervention with its associated shorter length of stay is likely to influence the total budget, and this may be of interest to decision makers. Detailed comparisons of such health care expenditure estimates across countries may also play a part in assessing the aggregate performance of health care systems [7]. Finally, the results of these studies can provide useful information to communicate the cost burden of a specific disease to a wider non-specialist audience [8].

A recent study has published detailed estimates of the costs of cerebrovascular diseases as part of a study of cardiovascular disease (CVD) related expenditures in the United Kingdom [9]. The authors estimated the annual healthcare costs for cerebrovascular diseases for the UK National Health Service (NHS) to be £5.2 billion and the total economic burden (including non-health care costs) to be £8.5 billion in 2004. aSAH has been estimated to be around 3% of all annual cerebrovascular events in the Oxfordshire region [10]. Although it is tempting to apply this figure to the cerebrovascular disease costs already calculated to estimate the UK costs of aSAH, this could produce seriously flawed results if applied generally: cerebrovascular diseases encompass different types of conditions and it is likely that each of them has different resource use consumption patterns; e.g. length of stay varies across cerebrovascular conditions. As a result, to calculate reliable cost estimates of aSAH we need to undertake a specific cost-of-illness study.

The main objectives of this study were to estimate the economic costs of aSAH in the United Kingdom, including direct healthcare costs, informal care costs and employment-related costs. The number of life-years and quality-adjusted life years (QALYs) lost due to premature death are also reported.

## Methods

### Methodological background

A cost-of-illness study was designed to identify, measure and value all resources related to aSAH [11]. The perspective adopted in this study was societal. Health care costs directly related to the NHS and non-healthcare costs associated with unpaid care and productivity losses from morbidity or premature death were considered.

A prevalence-based approach was adopted, where all costs related to aSAH in 2005 were measured regardless of the date the haemorrhage occurred. Health care costs were adjusted to 2005 UK prices using the Hospital and Community Health Services inflation index [12]. Non-healthcare costs were also expressed in 2005 prices.

Aggregate national data on morbidity, mortality, resource use and disease-related costs were available and therefore a top-down costing method was used in this study. Epidemiological and resource use data were available from several sources [9,13-16]. Population ratios were used to adjust to UK levels when data only covered England or England and Wales. To complete the information not available at a national level, data from the International Subarachnoid Aneurysm Trial (ISAT) were used: patients recruited to this large trial were broadly representative of the UK aSAH population in terms of age, geographical distribution, severity and other characteristics [17].

### The epidemiology of aSAH

To estimate the number of patients with aSAH in 2005, the total number of admissions in the UK, as reported in Hospital Episode Statistics (HES), was divided by the number of admissions each patient incurred. The number of admissions includes both new cases of aSAH and any re-admissions following episodes in previous years. The number of admissions each patient incurred was assumed to be similar to the information from the ISAT dataset where each patient incurred on average 1.07 admissions during the first year after the haemorrhage.

### Life-years (LYs) and quality-adjusted life years (QALYs)

Sex and age-specific data on mortality due to aSAH (ICD-10 code I60) and all-cause mortality data were available from the Office for National Statistics (ONS) [18]. Sex and age-specific abridged life tables were generated for a general population cohort using all-cause mortality excluding aSAH, and for an aSAH cohort using aSAH specific-mortality [19]. A hypothetical cohort of 1,000 individuals in 11 age bands by gender was defined. The number of persons at the beginning of each interval was calculated by subtracting from the number of people in the previous interval the number of deaths occurring in that interval. The number of person-years in each interval was calculated assuming that deaths occurred in the mid-point of the interval and adjusting for the length of the interval. The cumulative person-years were estimated as the number of person-years in an interval plus any previous year. These were then divided by the number beginning in each interval to estimate life expectancy in each age group. Quality-adjusted life-years (QALYs) in each age interval were calculated by multiplying the number of persons-years by an estimate of health-related quality of life (HRQL) in that interval. Quality-adjusted life expectancy (QALE) was computed similarly to life expectancy but using cumulative QALYs as the numerator.

HRQL was extracted from the EuroQol EQ-5D instrument [20]. The EQ-5D is a generic health outcome that measures quality of life widely used in the economic evaluation of health care technologies. It includes five domains with three possible levels in each domain. Health states from the EQ-5D can be converted into a utility value using a validated tariff estimated with time trade-offs methods in a large representative British sample [21]. EQ-5D population norms for the general population cohort and EQ-5D data at one year follow-up from the ISAT study for the aSAH cohort were used in the life table approach [17,21]. The same HRQL was assumed for age bands <1-9 as for 10-19, and for those over 79 as for 70-79. LYs and QALYs in each cohort were calculated by multiplying the aSAH population by the corresponding life expectancy and QALE. Differences in LYs and QALYs were computed by subtraction between the all cause and disease specific (aSAH) cohorts.

### Healthcare expenditure

Hospital inpatient admissions, operations for aSAH, cerebrovascular rehabilitation, accident and emergency care, hospital day cases, hospital outpatient care, primary care and community health and social services (CHSS) were the healthcare expenditure categories included.

Volumes of aSAH-related resources in each category were extracted from the sources available and multiplied by the appropriate unit costs. Unit costs were obtained from NHS reference costs, standard national publications and a recent study of the UK costs of endovascular and surgical clipping following aSAH [5,22,23].

Medication costs were not included in this study as their contribution to overall aSAH costs is expected to be very small. In addition, medical negligence and private healthcare costs were not included in the analysis due to lack of data availability.

#### Hospital inpatient admissions

Inpatient admissions consist of aSAH-related bed days in NHS hospitals, where aSAH is recorded as the primary reason for the admission. The number of inpatient bed days for England was extracted from the Hospital Episode Statistics and adjusted to UK levels.

#### Operations for aSAH

A recent study of Neurosurgical Units in the UK and Ireland reported that 2198 out of 2397 (91.7%) patients with a confirmed ruptured aneurysm received a repair procedure[24]. This proportion was applied to the estimated total number of UK aSAH patients to calculate the number of patients treated.

#### Accident and emergency care

Accident and emergency care consists of all aSAH-related hospital emergency visits. Data for England were obtained from the Hospital Episode Statistics database and adjusted to UK levels.

#### Hospital day cases and hospital outpatient care

This category includes the number of day cases and outpatient care in the form of follow up angiograms performed on patients. The proportion of patients attending for a follow up angiogram was extracted from the ISAT study and was estimated to be 42%.

#### Cerebrovascular rehabilitation

The number of patients completing cerebrovascular rehabilitation programmes was calculated as the product of the number of patients with aSAH and the proportion of those patients attending a rehabilitation programme. The proportion of patients attending a rehabilitation programme was extracted from the ISAT study and was estimated to be 7%.

#### Primary Care

Primary care consultations consist mainly of visits to a general practitioner at a surgery. Consultations were obtained from a large national survey performed in England and Wales[15], and estimates were then adjusted to UK levels.

#### Community Health and Social Services (CHSS)

All health and social care provided in the community including professional advice and support, general patient care and other healthcare services provided were included in this category. The cost of CHSS attributable to aSAH was calculated as a proportion of the total CHSS spending for cerebrovascular diseases in the United Kingdom. The total UK CHSS costs for cerebrovascular diseases were taken from the Department of Health Burden of Disease publication updated to 2005 prices, [14]. Results from the Oxford Vascular Study (OXVASC) suggest that 3% of all annual cerebrovascular events in the Oxfordshire region were aSAH and therefore this was the baseline proportion applied to total CHSS expenditure on cerebrovascular diseases in this analysis [10].

### Non-healthcare expenditure

#### Informal care

Informal care costs were measured as the monetary value of time spent by carers while providing care for relatives with aSAH (the opportunity costs of unpaid care). Routine databases on informal care for cerebrovascular diseases are not available yet, and researchers have estimated these costs using different methods. Luengo-Fernandez et al. estimated the informal care costs of cerebrovascular diseases in the United Kingdom using European and national sources [9,25-28]. They extracted information on the proportion of care given by working age carers, the number of hours spent caring and the number of informal carers in each age group. They valued informal care costs using wage rates for the employed carers (economically active) and minimum wages for retired or unemployed carers (economically inactive) [29,30]. The costs of informal care attributable to aSAH were assumed to be 3% of all informal care costs for cerebrovascular diseases, in line with findings from the OXVASC study [10].

#### Productivity losses

Productivity costs were estimated as the earnings lost as a result of aSAH-related mortality and morbidity.

Productivity loss from aSAH-related death was calculated as the product of age and sex specific mortality deaths and the number of working years lost due to premature death. The age and sex specific mortality rates due to aSAH were extracted from the ONS Mortality Statistics [18]. Working years lost were adjusted to take into account future changes in the size of the labour force using economic activity data [16]. This product was then multiplied by average annual earnings [29]. The number of future working years lost due to premature death in 2005 was used as a proxy for premature deaths in previous years. As this is a prevalence study no discounting was applied.

Productivity losses due to aSAH-related morbidity were calculated using both the human capital and the friction method approaches [31]. The first method estimates losses as the product of the number of days off work and average daily earnings. Information on absence from work of patients with aSAH was extracted from the ISAT dataset. In the friction method approach it is assumed that absent workers are likely to be replaced by other workers within some period of time - the friction period. This period was assumed here to be 90 days [32]. The friction-period adjusted morbidity loss was estimated by multiplying the unadjusted productivity loss (human capital approach) by the friction period and then dividing this product by the age and sex specific duration of incapacity spells, which was extracted from the ISAT dataset.

### Sensitivity analysis

The impact of varying the number of admissions each patient incurred during the first year after the haemorrhage extracted from the ISAT study and its impact in the LYs and QALYs lost results was also evaluated. A threshold of a 20% change in the parameter was used.

To test how changes in key resource estimates, unit costs, informal care and productivity costs affect the baseline results, one-way sensitivity analysis was performed. Only those parameters informing resource categories that contributed substantially to the overall and NHS costs were included in the sensitivity analysis. The effect of 20% changes on each parameter was evaluated. The impact of altering the proportion of cerebrovascular diseases attributable to aSAH from 3% to 1% or 6% was also evaluated.

The 20% threshold was used to maintain comparability and consistency across cost-of-illness studies in the area of cerebrovascular diseases [9,33].

## Results

### The epidemiology of aSAH in the United Kingdom

The number of hospital admissions due to aSAH was estimated to be 7,727 (2,962 men and 4,765 women) in the United Kingdom in 2005. Applying the baseline estimate of 1.07 admissions per patient on average, 7,221 (2,768 men and 4,453 women) patients were estimated to have aSAH in the United Kingdom in 2005.

Life expectancy and QALE for the general population and the aSAH cohorts by gender and age group are reported in tables 1 and 2 respectively. Table 3 suggests that the number of life years and quality adjusted life years lost as a result of aSAH when compared to the life experience of the general population was 80,356 LYs and 74,807 QALYs respectively; dividing by the annual number of aSAH cases, this gives an average loss per case of 11.1 life years and 10.4 quality adjusted life years.

**Table 1:** Life expectancy and QALE in the general population life table cohort

Age interval	Probability of death in interval	Number beginning interval	Person-years in interval	Cumulative person-years	Life expectancy	HRQL (EQ5D)	QALYs person-years	Cumulative QALYs person-years	QALE
Males									
<1-9	0.007	1000	9964	76618	76.6	0.94	9366	66817	66.8
10-19	0.003	993	9913	66654	67.1	0.94	9319	57451	57.9
20-29	0.008	990	9861	56741	57.3	0.935	9220	48132	48.6
30-39	0.012	982	9766	46880	47.7	0.92	8985	38912	39.6
40-49	0.024	971	9593	37114	38.2	0.875	8393	29927	30.8
50-59	0.058	948	9201	27521	29.0	0.81	7453	21534	22.7
60-69	0.149	893	8261	18320	20.5	0.78	6443	14081	15.8
70-79	0.362	760	6219	10059	13.2	0.765	4758	7638	10.1
80-89	0.715	484	3112	3840	7.9	0.75	2334	2880	5.9
90-99	0.955	138	722	728	5.3	0.75	542	546	4.0
100+	1	6	6	6	1.0	0.75	5	5	0.8
									
Females									
<1-9	0.006	1000	9970	80908	80.9	0.94	9372	70133	70.1
10-19	0.002	994	9932	70938	71.4	0.94	9336	60761	61.1
20-29	0.003	992	9908	61005	61.5	0.935	9264	51424	51.8
30-39	0.006	989	9862	51097	51.7	0.92	9073	42160	42.6
40-49	0.016	983	9756	41235	41.9	0.88	8585	33087	33.7
50-59	0.038	968	9496	31479	32.5	0.83	7882	24502	25.3
60-69	0.095	931	8872	21983	23.6	0.795	7054	16620	17.8
70-79	0.253	843	7364	13111	15.5	0.745	5486	9566	11.3
80-89	0.604	630	4396	5746	9.1	0.71	3121	4080	6.5
90-99	0.932	250	1333	1350	5.4	0.71	946	958	3.8
100+	1	17	17	17	1.0	0.71	12	12	0.7

HRQL: health-related quality of life; EQ-5D: EuroQol 5D instrument; QALE: quality-adjusted life expectancy; QALY: quality-adjusted life years

**Table 2:** Life expectancy and QALE in the aSAH life table cohort

Age interval	Probability of death in interval	Number beginning interval	Person-years in interval	Cumulative person-years	Life expectancy	HRQL (EQ5D)	QALYs person-years	Cumulative QALYs person-years	QALE
Males									
<1-9	0.187	1000	9037	39207	39.2	0.68	6163	27791	27.8
10-19	0.179	807	7343	30170	37.4	0.68	5008	21628	26.8
20-29	0.123	661	6181	22827	34.5	0.74	4593	16620	25.1
30-39	0.160	575	5264	16646	28.9	0.70	3706	12027	20.9
40-49	0.211	478	4227	11382	23.8	0.72	3039	8321	17.4
50-59	0.221	368	3187	7155	19.5	0.76	2406	5282	14.4
60-69	0.211	270	2254	3968	14.7	0.74	1660	2875	10.7
70-79	0.399	181	1252	1715	9.5	0.71	887	1215	6.7
80-89	0.423	69	404	463	6.7	0.71	286	328	4.7
90-99	0.536	11	58	59	5.1	0.71	41	41	3.6
100+	1	0	0	0	1.0	0.71	0	0	0.7
									
Females									
<1-9	0.361	1000	8175	35686	35.7	0.83	6745	27417	27.4
10-19	0.051	635	6182	27511	43.3	0.83	5101	20672	32.6
20-29	0.139	601	5589	21329	35.5	0.83	4611	15571	25.9
30-39	0.158	516	4743	15740	30.5	0.69	3265	10960	21.2
40-49	0.189	432	3889	10996	25.4	0.69	2665	7695	17.8
50-59	0.214	345	3032	7108	20.6	0.71	2143	5030	14.6
60-69	0.256	261	2185	4075	15.6	0.72	1582	2887	11.1
70-79	0.349	176	1307	1890	10.7	0.69	902	1305	7.4
80-89	0.549	85	504	583	6.8	0.69	348	402	4.7
90-99	0.592	15	79	79	5.2	0.69	54	54	3.6
100+	1	0	0	0	1.0	0.69	0	0	0.7

HRQL: health-related quality of life; EQ-5D: EuroQol 5D instrument; QALE: quality-adjusted life expectancy; QALY: quality-adjusted life years

**Table 3:** Life-years (LYs) and quality-adjusted life years (QALYs) lost in the aSAH cohort compared to the general population cohort

Age interval	aSAH population	Life years aSAH cohort (1)	Life-years general population cohort (2)	Difference (2)-(1)	QALYs aSAH cohort (3)	QALYs general population cohort (4)	Difference (4)-(3)
Males							
<1-9	15	569	1111	543	403	969	566
10-19	46	1710	3071	1362	1226	2647	1422
20-29	114	3930	6524	2595	2861	5534	2673
30-39	339	9818	16188	6370	7094	13437	6343
40-49	598	14250	22862	8612	10417	18435	8018
50-59	687	13375	19963	6587	9874	15620	5747
60-69	536	7881	10993	3112	5711	8450	2739
70-79	271	2568	3591	1023	1820	2726	907
80-89	147	982	1168	186	696	876	180
90-99	16	80	82	2	57	62	5
100+	15	0	0	0	0	0	0
							
LYs or QALYs lost males				30391			28599
							
Females							
<1-9	4	159	361	202	122	313	191
10-19	38	1644	2707	1064	1235	2319	1084
20-29	87	3087	5350	2264	2253	4510	2257
30-39	333	10136	17176	7039	7058	14172	7113
40-49	842	21427	35332	13905	14994	28350	13356
50-59	1,186	24410	38575	14165	17274	30025	12750
60-69	904	14106	21335	7228	9992	16130	6138
70-79	705	7576	10965	3389	5230	8001	2771
80-89	301	2054	2749	696	1418	1952	534
90-99	52	271	284	13	187	201	14
100+	4	0	0	0	0	0	0
							
LYs or QALYs lost females				49964			46208
							
**Total LYs or QALYs lost**				**80356**			**74807**

LYs: life-years; QALY: quality-adjusted life years; aSAH: aneurysmal subarachnoid haemorrhage

### Healthcare costs

Table 4 shows a summary of the results of the NHS cost categories. Aneurysmal subarachnoid haemorrhage cost the NHS £168.2 million with a cost per patient estimated to be £23,294 in 2005. Hospital inpatient care accounted for 59% of the estimated costs with 123,968 inpatient bed days and associated costs of £98.7 million. The second largest component with 18% of the overall aSAH costs was Community Health and Social Services which accounted for £30.2 million. aSAH operations cost the NHS £25.4 million with 6,625 patients receiving a repair procedure. Cerebrovascular rehabilitation costs were estimated to be £10.6 million with 506 patients spending 47,540 days at a rehabilitation clinic accounting for 6% of the health care costs. Accident and emergency, hospital day cases, hospital outpatient care and primary care cost the NHS £3.2 million in 2005.

**Table 4:** Summary of costs of aneurysmal subarachnoid haemorrhage in the UK in 2005

Type of resource used	Unit of measurement	Units of resources consumed	Average unit cost (£2005)	Total cost (£2005 million)	Sources of data (reference number)
**Health care cost**					

Hospital inpatient care	Inpatient bed days	123,968	£796	£98.7	6

Surgical operations for aSAH	Operated patients	6,625	£3,833	£25.4	6

Cerebrovascular rehabilitation	Days at rehabilitation clinic	47,540	£224	£10.6	6,10

Accident and emergency	Attendances	5,140	£106	£0.5	6

Hospital day case and outpatient care	Day cases	3,049	£691	£2.1	6

Primary care	Doctor consultations at clinic	20,370	£30	£0.6	8

Community health/social services				£30.2	3,7,10

					

**Health care cost subtotal**				**£168.2**	

					

**Non-health care cost**					

Hours of informal care	Hours of caring by economically active carers per year	3,311,769	£9	£31.4	2,3,18,19,20,21

	Hours of caring by economically inactive carers per year	2,080,325	£5	£10.5	2,3,18,19,20,21

**Informal care cost subtotal**				**£41.9**	

					

Productivity loss					

Mortality	Working years lost (men)	7,564	£25,100	£152.7	6,9,10,22

	Working years lost (women)	9,088	£19,400	£126.2	6,9,10,22

Morbidity	Certified incapacity days (men)	122,280	£85	£10.4	6,10,23

	Certified incapacity days (women)	210,112	£51	£10.8	6,10,23

Morbidity (Friction adjusted) men				£3.2	6,10,23,24

Morbidity (Friction adjusted) women				£3.1	6,10,23,24

					

**Productivity loss subtotal**				**£300.1**	

(Friction adjusted)				**£285.2**	

					

**Non-health care subtotal**				**£342.0**	

(Friction adjusted)				**£327.1**	

					

**Total economic burden**				**£510.2**	

(Friction adjusted)				**£495.3**	

aSAH: aneurysmal subarachnoid haemorrhage

### Non-healthcare costs

#### Informal care costs

Table 4 shows that society spent 3.3 million hours of caring by economically active carers and 2 million hours of caring by economically inactive carers. The total informal care costs due to aSAH were estimated to be £41.9 million.

#### Productivity costs

Table 4 also reports the productivity costs associated with aSAH. A total of 7,564 working years were lost by men with future forgone earnings calculated at £152.7 million. Females lost 9,088 years and associated future forgone earnings were estimated to be £126.2 million.

The total number of certified incapacity days was estimated to be 122,280 for males and 210,112 for females. Morbidity costs were £21.2 million overall, however when adjusting for the friction period the cost was estimated to be £6.3 million.

The total economic burden of aSAH in the United Kingdom was estimated to be £510 million using the human capital approach for morbidity costs and £495 million when using the friction method.

### Sensitivity analysis

Reducing the number of admissions per patient per year a 20%, increased the number of life-years and quality-adjusted life years lost to 86,386 and 83,531 respectively. If the same parameter is increased a 20%, the number of life-years and quality-adjusted life years lost was estimated to be 75,056 and 68,088 respectively.

**Figure 1:**
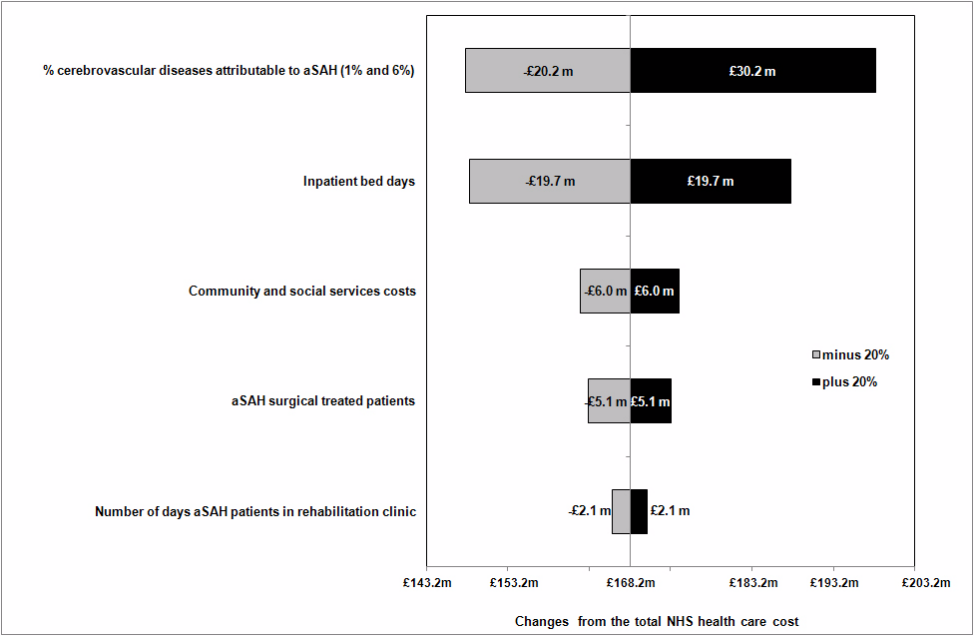
Sensitivity of National Health Service (NHS) aSAH-related costs to ± 20% changes in key factors

Figure 1 shows how sensitive the main estimate of NHS healthcare costs was to different assumptions concerning resource use or unit costs, holding everything else constant. For example, reducing the number of bed days to 99,174, that is a 20% reduction, decreased total NHS healthcare costs by 12%. Similarly, if we increase the proportion of all cerebrovascular diseases associated to aSAH to 6% (this parameter affects community and social service costs), NHS healthcare costs increases by 18%.

**Figure 2:**
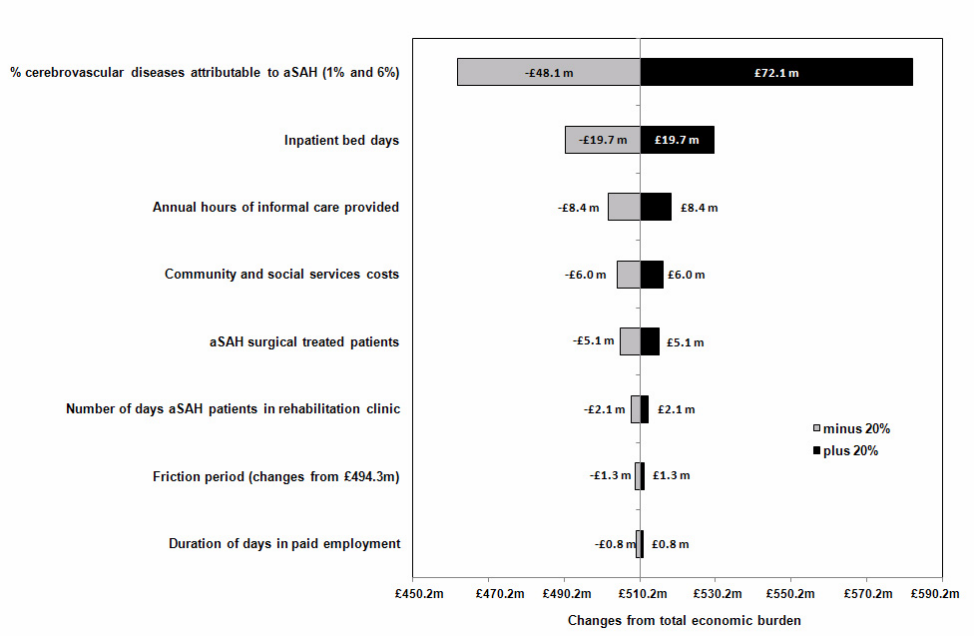
Sensitivity of aSAH disease-related costs to ± 20% changes in key factors

Figure 2 shows how sensitive total aSAH-related costs (including non-healthcare costs) were to changes in key factors holding everything else constant. Overall, changes in the proportion of all cerebrovascular diseases associated with aSAH, and the number of inpatient bed days, had the greatest impact on aSAH-related costs with changes of 16% and 5% respectively.

## Discussion

This paper reports the first cost-of-illness analysis of aSAH in the United Kingdom. Aneurysmal subarachnoid haemorrhage cost the NHS £168.2 million in 2005 with overall aSAH-related costs estimated to be £510 million using the human capital and £495 million when using the friction method. This accounts for 6% of the £8.8 billion (2005 prices) for the UK costs of all cerebrovascular diseases reported by Luengo-Fernandez et al in 2004 [9], and so constitutes a significantly greater proportion of total CVD expenditure than of CVD events: Rothwell et al estimated that 3% of CVD events in Oxfordshire from 2002 to 2005 were aSAH [10].

Age and sex-specific life expectancy and quality-adjusted life expectancy for a general population and an aSAH cohort are reported in this study, indicating that aSAH is associated with a loss of 11.1 years of life expectancy and 10.4 quality adjusted years of life expectancy compared to a general population. Quality of life of aSAH patients has also been reported recently in a study using the Short-Form SF-36 but no utility values to derive QALYs were included in this analysis [34]. Therefore to our knowledge no similar estimates, as detailed as the current research, have been reported to date. The SF-36 is a multiattribute generic quality of life outcome widely used by the clinical community [35]. It includes 36 items that can be summarised in eight domains plus a physical and a mental component.

This study estimated the per patient healthcare costs of treating aSAH to be £23,294 per annum. A recent detailed cost study of the UK costs of endovascular and surgical clipping following aSAH has reported that these patients cost the NHS £19,306 (community and social service costs not included in the study) on average during the first year after the collapse [5]. Removing CHSS from the cost estimates presented here, the NHS cost per patient would be £19,107; this is similar to the result reported by the recent UK cost study and supports the figures presented here.

Several limitations of this study need to be highlighted. The community and social service costs extracted from the Burden of Disease Report for this study are becoming out of date [14]. The recent primary care trusts programme budget is beginning to provide more reliable estimates of these cost figures [36]. However, no detailed data on aSAH were available from this new source when performing this study. The characteristics and management of patients in a clinical trial such as ISAT may differ from routine clinical care and hence the parameters used in this study may be subject to some degree of bias. For example, the proportion of patients attending a follow-up angiogram or a rehabilitation programme was extracted from ISAT and therefore refers only to treated aneurysms. The same proportions for untreated aneurysm were not available when conducting this study. However, the sensitivity analysis showed the effect of varying these parameters on the overall costs. Finally, the one-way sensitivity analysis performed ignores any possible covariance across different categories of costs and hence this aspect needs to be considered in future research.

An additional limitation of the current research was the ability to include co-morbidity costs related to aSAH. Aggregate data on finished admissions where aSAH was the primary diagnosis was the main source used in the calculation of the hospital inpatient admission costs and the number of patients with aSAH. If co-morbidities costs are substantial our results may be sensitive to this parameter.

Cost-of-illness studies have been criticised for the variety of methods applied to report their results. This reflects the fact that clear guidelines on how to conduct these analyses are not currently available [37]. This research mainly uses aggregate data coded by specific aSAH diagnosis to minimise the bias of including potential costs not related to the disease. It can be argued that using this type of data from national databases is subject to confounding across health areas. Nevertheless, the type of health care received by aSAH patients is very specific and therefore the impact of confounding on the overall costs estimated is expected to be limited. In addition, cost-of-illness studies are systematically different from traditional methods of economic evaluation and therefore the results from such studies cannot be interpreted in the same manner. This has received some criticism from the health economics community and although this is partly true, cost-of-illness studies provide useful information to prioritise healthcare. Cost-of-illness studies provide information that may be useful to decision makers when identifying priority disease areas for research funding and to develop prevention policies [11]. In addition, these studies provide a framework to evaluate the impact of population changes such as ageing on health outcomes and overall healthcare costs. Finally, the results reported here will provide useful information with which to populate economic models of interventions in the field of aSAH.

## Conclusion

The economic and disease burden of aSAH in the United Kingdom is reported in this study. Decision-makers can use these results to complement other information when informing prevention policies in this field and to relate health care expenditures to disease categories. In addition, the results from this study will inform future epidemiological and economic models with useful data on quality of life and costs of patients with aSAH.

## List of abbreviations

aSAH: Aneurysmal subarachnoid haemorrhage; CHHS: Community Health and Social Services; CVD: Cardiovascular disease; EQ-5D: EuroQol 5 dimension instrument; HES: Hospital Episode Statistics; HRQL: Health-related quality of life; ICD-10: International Classification of Diseases 10th revision; ISAT: International Subarachnoid Aneurysm Trial; LYs: Life-years; NHS: National Health Service; ONS: Office for National Statistics; OXVASC: Oxford Vascular Study; QALE: Quality-adjusted life expectancy; QALYs: Quality-adjusted life years; UK: United Kingdom.

## Competing interests

This research was supported by an unrestricted grant from Actelion Pharmaceuticals Ltd. Oliver Rivero-Arias is funded by a Researcher Development Award from the Department of Health and NHS R&D. The Health Economics Research Centre receives some of its funding from the National Institute of Health Research. The authors report no conflict of interest.

## Authors' contributions

ORA revised the original project proposal, collected, analysed and interpreted the data and wrote the main draft of the manuscript. AG wrote the original project proposal, supervised the main analysis and revised the manuscript. JW revised the original project proposal, supervised the main analysis and revised the manuscript. All authors read and approved the final manuscript.
